# The Use of Probiotic Therapy to Modulate the Gut Microbiota and Dendritic Cell Responses in Inflammatory Bowel Diseases

**DOI:** 10.3390/medsci7020033

**Published:** 2019-02-22

**Authors:** Pablo Alagón Fernández del Campo, Alejandro De Orta Pando, Juan Ignacio Straface, José Ricardo López Vega, Diego Toledo Plata, Sebastian Felipe Niezen Lugo, Diego Alvarez Hernández, Tomás Barrientos Fortes, Laila Gutiérrez-Kobeh, Sandra Georgina Solano-Gálvez, Rosalino Vázquez-López

**Affiliations:** 1 Departamento de Microbiología del Centro de Investigación en Ciencias de la Salud (CICSA), FCS, Universidad Anáhuac México Campus Norte, Cuidad de México 52786, Mexico; pabloalagon@hotmail.com (P.A.F.d.C.); call.alexdopl@gmail.com (A.D.O.P.); juan.straface@windowslive.com (J.I.S.); jrlv_jrlv@yahoo.com.mx (J.R.L.V.); a01362487@gmail.com (D.T.P.); sebastiannl7@hotmail.com (S.F.N.L.); diego.alvarez.hernandez@hotmail.com (D.A.H.); 2 Director Facultad de Ciencias de la Salud, Universidad Anáhuac México, Cuidad de México 52786, Mexico; tbarrien@anahuac.mx; 3 Unidad de Investigación UNAM-INC, División Investigación, Facultad de Medicina, Universidad Nacional Autónoma de México-Instituto Nacional de Cardiología “Ignacio Chávez”, Mexico City 14080, Mexico; lgutierr@unam.mx; 4 Departamento de Microbiología y Parasitología, Facultad de Medicina, Universidad Nacional Autónoma de México, Ciudad de México 04510, Mexico; solano-sandra@hotmail.com

**Keywords:** inflammatory bowel disease, dendritic cells, ulcerative colitis, Crohn’s disease, gut microbiota, probiotic

## Abstract

Recent investigations have shown that different conditions such as diet, the overuse of antibiotics or the colonization of pathogenic microorganisms can alter the population status of the intestinal microbiota. This modification can produce a change from homeostasis to a condition known as imbalance or dysbiosis; however, the role-played by dysbiosis and the development of inflammatory bowel diseases (IBD) has been poorly understood. It was actually not until a few years ago that studies started to develop regarding the role that dendritic cells (DC) of intestinal mucosa play in the sensing of the gut microbiota population. The latest studies have focused on describing the DC modulation, specifically on tolerance response involving T regulatory cells or on the inflammatory response involving reactive oxygen species and tissue damage. Furthermore, the latest studies have also focused on the protective and restorative effect of the population of the gut microbiota given by probiotic therapy, targeting IBD and other intestinal pathologies. In the present work, the authors propose and summarize a recently studied complex axis of interaction between the population of the gut microbiota, the sensing of the DC and its modulation towards tolerance and inflammation, the development of IBD and the protective and restorative effect of probiotics on other intestinal pathologies.

## 1. Background

Inflammatory bowel disease (IBD) is comprised of a group of pathological entities characterized by inflammation of the small intestine and colon. The two main diseases relative to IBD are ulcerative colitis (UC) and Crohn’s disease (CD) [1] In the population younger than 20 years of age, the incidence of CD amounts to approximately 43 out of 100,000 inhabitants whereas that of UC amounts to 28 out of 100,000. [2]. Given these are chronic diseases there is an incidence increase seen in patients older than 20 years of up to 201 out of 100,000 for CD and 238 out of 100,000 for UC. [2,3]. The highest incidence rates and prevalence of Crohn’s disease and ulcerative colitis are predominantly reported in industrialized countries such as northern Europe, the United Kingdom and North America. These rates have reached a plateau after the steady rise seen in these regions after the end of World War II, while rates continue to rise in low-incidence areas such as southern Europe, Asia and most developing countries [4,5,6]. In the industrialized countries, the incidence rates range from 6.5 to 16.0 cases per 100,000 persons/year, while the prevalence rates range from 26 to 214 patients per 100,000 persons/year [7]. Within these countries, the United States has a prevalence range for UC of 37 to 246 cases per 100,000 persons and an incidence range of 2.2 to 14.3 cases per 100,000 per persons/year. For CD, the prevalence ranges from 26 to 199 cases per 100,000 persons and the incidence ranges from 3.1 to 14. 6 cases per 100,000 persons/year [8]. In Europe, UC has incidence rates range from 1.5 to 20.3 cases per 100,000 person/year, while these rates range from 0.7 to 9.8 cases per 100,000 person-years for CD [4].

One of the developing regions that continues its rise in incidence and prevalence of IBD is Latin America [4]. Still, epidemiologic studies in the countries within Latin America are scarce due to the gradual onset, the lack of universally accepted criteria for diagnosis and the idea that in the past this disease was rare there [7]. Most of these countries do not have an efficient data recording method in order to provide information for epidemiologic studies. However, some have data like Colombia, Peru and Brazil. In Colombia, from 2001 through 2009, 202 cases were diagnosed with IBD, where 80.7% of them had UC and 15.8% had CD [8]. Brazil has made few studies, usually only describing clinical aspects of the patients that arrive at the hospitals in the region with no incidence and prevalence. The most recent data has only proven that CD is more prevalent than UC [7]. In Peru, several studies on UC have been made in hospitals like the Hospital Guillermo Almenara which received 74 cases in 52 years, the Hospital Edgardo Rebagliati which received 43 cases in 2 years and the Hospital Cayetano Heredia which received 27 cases in 7 years. For CD, 17 cases were reported in a period of 20 years in the Hospital Edgardo Rebagliati [8]. Regardless, epidemiologic data is minimum in other countries of Latin America, including Mexico.

## 2. Microbiota–Dendritic Cell-Mucosal Immune Response–IBD Interaction

The causes leading to the development of IBD are still up to this date uncertain. Regardless, it has been proposed that its origin could be multifactorial, involving the patient’s genetic predisposition, nutrition and eating habits, as well the status of intestinal microbiota and the integrity of the intestinal barrier function [9,10]. The interaction of all these factors has effect on both the intestinal homeostasis and the pathological condition of the uncontrolled immune-mediated inflammatory response present in IBD.

**Figure 1:**
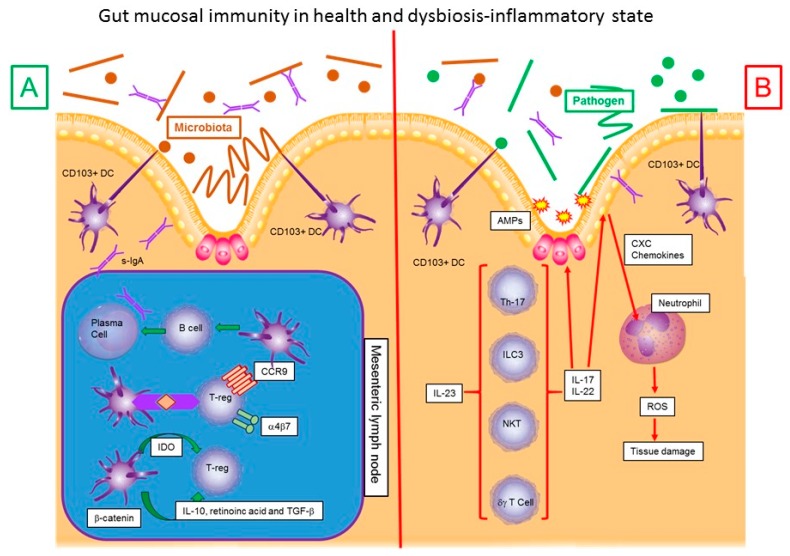
Gut mucosal immunity in health and dysbiosis-inflammatory state schematic representation of the interaction between gut microbiota, dendritic cells and inflammatory response in Gut mucosa in a healthy state (**A**) and gut mucosa in dysbiotic—inflammatory state (**B**). Taken from [11]. α4β7: α4β7 Integrin, AMPs: antimicrobial peptides, CCR9: C-C chemokine receptor type 9, CXC: CXC Chemokines family, DC: Dendritic Cell, IDO: Indoleamine-pyrrole 2,3-dioxygenase, IL-10: Interleukin 10, IL-17:Interleukin 17, IL-22: Interleukin 22, IL-23: Interleukin 23, ROS: Reactive oxygen species, s-IgA: Secretory Immunoglobulin A, TGF-β: Transforming growth factor beta, T-reg: Regulatory T cells, Th-17: T-helper cell 17 lineage, NKT: Natural killer T cells.

It all in fact originates in the lamina propria (LP) of a healthy intestine, where dendritic cells (DC) sense antigens, which originate from food and bacteria that make up the intestinal microbiota as well as of its metabolite. The count of bacteria and the microbiota is made by the presence of various receptors, made up mainly by different types of toll like receptors (TLR) (Figure 1) [11]. Dendritic cells are surveillance cells that among other tasks, play the indispensable function of distinguishing between self and non-self. They have the capacity to recognize different molecules such as proteins, lipids, carbohydrates and nucleic acids of bacterial, viral, fungal or protozoan origin known as pathogen-associated molecular patterns (PAMPs). To achieve this surveillance task, DCs possess distinct types of receptors among which are: TLR, RIG-I-like receptors (RLR), NOD like receptors (NLR) and C-type lectin receptors (CLR) [12].

One of the most important events for keeping intestinal homeostasis is the induction by DCs of anergic and/or regulatory T cells (Tregs), which is crucial for the maintenance of peripheral tolerance in addition to regulating the response by altering the Th1/Th2/Th17 balance. The adequate induction of tolerance by DCs depends on various factors such as the state of maturation, the DC subsets, the exposure to anti-inflammatory, immunosuppressive, environmental or microbial stimuli, among others [13,14,15,16,17]. In relation to the state of maturation of DCs in the induction of tolerance, immature DCs characterized by a low expression of surface major histocompatibility complex class II (MHC II ) and costimulatory molecules, induce suboptimal T-cell priming. Immature DCs promote tolerance in vivo by either deleting antigen-specific T cells or by expanding regulatory T cells [18,19,20,21].

On the other hand, mature DCs promote immunogenic responses [22,23,24], although under some conditions these DCs can be tolerogenic as has been demonstrated with the disruption of E-cadherin-mediated DC-DC interaction that promotes DC maturation and the secretion of high levels of IL-10 that induces the tolerogenic response [25].

The subtype of DC is another factor involved in the induction of tolerance and is influenced by the local environment and state of activation. As example of the former, in the intestine, various factors produced by the epithelial cells and stromal cells such as transforming growth factor beta (TGF-β) [26], Thymic stromal lymphopoietin (TSLP) [27] and retinoic acid (RA) [28] shape the functions of tolerogenic DCs and of the latter, in the resting steady state certain DC subsets have a propensity to induce tolerogenic T cells. In particular, the presence of tolerogenic DC subsets has great relevance at mucosal surfaces where the immune system needs to play a relevant dual role of maintaining tolerance to self-antigens and commensals and mounting strong immune responses to pathogens. Thus, the tolerogenic subsets in the mucosal compartment prevent excessive inflammation and immunity against commensals and food or environmental antigens. As just mentioned, RA is an important compound produced by DCs for the generation of Tregs. The conversion of vitamin A-derived retinol to RA is catalysed by retinaldehyde dehydrogenases (ALDHs), which are crucial enzymes in the induction of Tregs and only DCs that possess them can promote intestinal tolerance and homeostasis. It has been shown that both CD103^+^CD11b^+^ and CD103^+^CD11b^−^ DCs can produce RA and induce Foxp3^+^ Tregs in vitro [14,29]. Interestingly, the tolerogenic properties of these cells can change under some circumstances as has been shown in colitic mice where CD103^+^ DCs do not induce Foxp3^+^ Tregs and instead favour the production of IFN-γ-producing CD4^+^ T cells [30]. Also, the ratio of DCs:Tcells is important in the induction of tolerance.

Another important factor that influences the tolerogenic properties of DCs is the exposure to microbial products. In many cases this is achieved through the recognition of different microbial ligands by pattern recognition receptors (PRRs) such as TLRs and CLRs that induce Th2 or tolerogenic responses. In relation to TLRs it has been shown that yeast zymosan [31,32,33,34] or *Y. pestis* virulence factor Lcr [35] signal through TLR2-TLR6 in DCs and induce regulatory T cells. Also, it has been suggested that in pDCs TLR-9 activation induces indole amine 2, 3-Dioxygenase (IDO), which promotes differentiation of Tregs and suppresses T-cell responses [36,37].

As for CLRs, it has been shown that activation of DC-SIGN in DCs by different microbial compounds promotes Tregs-responses [38]. Examples of these are cell surface compounds of *Lactobacillus reuteri* and *L. casei*, Lewis antigens on lipopolysaccharide (LPS) from *Helicobacter pylori* [39] and lpA of *L. acidophilus* NCFM [40] that bind to dendritic cell-specific intercellular adhesion molecule-3-grabbing non-integrin (DC-SIGN) and induce IL-10 production and suppress T-cell effector responses. It has also been shown that activation of SIGNR-1 in lamina propria DCs selectively induces IL-10 expression and promotes the induction of Tr1 regulatory cells [41]. All types of galectins, surface, secreted and endogenous, are also important molecules in the promotion of tolerance. In particular, Galectin-1-mediated signals promote tolerance in DCs by inducing the expression of several regulatory molecules like signal transducer and activator of transcription 3 (STAT3), suppressor of cytokine signaling 1 (SOCS1) and histone deacetylase 11 (HDAC11) [42,43].

The interaction of DCs with other cells is another factor that contributes to their tolerogenic profile. As an example, the tolerogenic responses in the intestine are maintained through the concerted action of interleukin 10 (IL-10)-secreting macrophages and DCs and IL-10 is fundamental in the suppression of inflammation such as colitis. Also, the interaction of DCs with non-hematopoietic cells is important for the induction of T-regulatory cells in the intestine. It has been shown that intestinal epithelial cells (IECs) are important in conditioning the intestinal DCs to a tolerogenic state through the secretion of anti-inflammatory mediators such as TGF-β, RA or granulocyte-macrophage colony-stimulating factor (GM-CSF). In addition to ECs, stromal cells also play a critical role in conditioning DCs to a regulatory or tolerogenic state in various organs such as the liver, intestine, gut-associated lymphoid tissues (GALT) and spleen [44,45,46].

As previously mentioned, at mucosal surfaces the immune system has to mount an immune response to microbes and yet be tolerant to commensals. In particular, intestinal commensals play a critical role in shaping DCs functions and promoting tolerance [47,48,49]. The induction of tolerance by commensals can be through the induction of TSLP and TGF-β by IECs or by the promotion of T-regulatory cells. It has been shown that DCs cultured in the presence of IECs and Gram-positive commensal bacteria differentiate into IL-10-producing tolerogenic DCs [50]. Also, in germ-free mice the colonization with the human commensal *Bacteroides fragilis* induces the development of Foxp3^+^ T-regulatory cells [51]. The generation of T regs can also be promoted by commensals products as in the case of polysaccharide A (PSA) of *B. fragilis* that can convert CD4^+^ T cells into Foxp3^+^ T-regulatory cells that produce IL-10 during commensal colonization. Contrarily, some commensals can have the capacity to suppress T-regulatory cells and promote Th17 responses. It has been shown that colonization of the small intestine of mice with a segmented filamentous bacterium (SFB) induces the appearance of Th17 cells in the lamina propria [52]. In addition to the commensal bacteria, intestinal helminths can also promote T-regulatory cell differentiation by activating TGF-βR on the antigen-presenting cells [53].

## 3. Microbiota and Inflammatory Bowel Disease

Concerning the origin of pathologies involving these mechanisms like IBD, the etiology still remains largely unknown. Some studies proclaim that the cause is multifactorial, involving intestinal microbiota, nutrition and the patient’s genetic profile. Recent studies evidence that specifically, the impact on the stability of the population and metabolism balance has a direct relationship in the physiopathology of IBD.

By using ribosomal RNA (rRNA) sequencing, Frank et al. proved in 2007 that the bacterial population in patients with IBD is anomalous. Predominant phyla in the intestinal microbiota of healthy individuals are *Firmicutes* and *Bacteroidetes*; however, in patients with IBD there is a decrease of the bacterial population or dysbiosis, a substantial depletion of these phyla and a substitution by phyla *Actinobacteria* and *Proteobacteria* (alpha, beta and gamma) [54]. Another study realized in 2012 by Morgan et al. explains that the dysbiosis observed in IBD generates an alteration of metabolism that leads to oxidative stress and perturbed nutrient availability during tissue damage [55]. Gevers et al. in 2014, on the other hand found another cause, demonstrating that antibiotic use amplifies the microbial dysbiosis associated with CD [56].

Another factor associated with dysbiosis is the arrival of pathogenic microorganisms. There are reports indicating that bacteria such as *Clostridium difficile*, enterotoxigenic *Escherichia coli* (ETEC) and *Salmonella* spp may participate in the development of IBD [57]. In a study made by Satokari R. in 2015, rats inhabiting polluted environments with pathogenic bacteria developed IBD, while rats that lived in pathogen-free conditions never developed the disease, suggesting a possible participation of pathogenic microorganism in the physiopathology of this disease [58].

These and other research establish a major participation of the imbalance of the population (dysbiosis) of the intestinal microbiota on IBD physiopathology, antibiotics or infections may induce this dysbiosis. One of the main effects of these previously mentioned conditions is the generation of conditions with high concentrations of reactive oxygen species (ROS) at an intestinal level which will contribute to a more severe inflammatory process at an intestinal level.

Among the causes or triggers of Crohn’s disease, there has been an association between the integrity of the immune system and the patient’s microbiota, this association is of great interest because it starts through the genetic factors of each individual.

Genes that confer a susceptibility to DC have been found, such as the nucleotide-binding oligomerization domain-containing protein 2 (*NOD2* gene), whose function is an immune reaction to recognize a peptidoglycan found in the cell of bacterium, both gram positive and negative [59]. Swidsinski et al. found that in patients with mutations in the *NOD2* gene there is an increased number of bacteria adhered to the mucosa and a decrease in the transcription of interleukin-10, an anti-inflammatory cytokine [60].

Individuals with mutations in *NOD2* and Autophagy-related protein 16-1 (*ATG16L1*), a gene involved in the process of autophagy that confers susceptibility to CD, present alterations in the strains that make up the microbiota, decreased levels of *Faecalibacterium* and high levels of *Escherichia* [61]. Kang et al. found a decrease in diversity within *Firmicutes phylum*, this decrease has been associated with a temporary instability of the microbiota in both individuals with DC and UC [62].

*Enterobacteria* are particularly elevated in patients with CD and UC, especially *Escherichia coli*, which has been isolated from the ileus of individuals with CD through biopsies in several studies [63]. This increase in enterobacteria could indicate a preference for an inflammatory environment such as that of individuals with CD. In fact, a reduction in *Escherichia/Shigella* levels has been demonstrated in patients with IBD after administration of mesalazine, an anti-inflammatory drug.

A second group of bacteria adhered to the mucosal layer of the colon in patients with CD and UC is *Fusobacterium*, gram-negative anaerobic bacteria that mainly colonize the oral cavity. *Fusobacterium* species raised in colorectal cancer biopsies have been found, this is of interest because IBD is one of the most important risk factors for the development of colorectal cancer, suggesting an association between these two diseases [64].

Unlike CD, UC has a less extensive description of the dysbiosis caused by it. In 1988 Tysk et al. found that UC seems to be more related to environmental factors than CD [65]. Spehlmann et al. found in 2008 that monozygotic twins with CD have a concordance of 30% whereas those with UC have one of only 10% [66].

A quantitative and qualitative decrease in the Firmicutes phylum has been shown to be present in both UC and CD. This includes many butyrate producing bacteria such as *Faecalobacterium prausnitzii* [67]. UC has shown a decrease of the Firmicutes and Bacteroides phylum while having an increase of the Proteobacteria and Actinobacteria phylum [68,69,70].

In a cohort conformed by 127 patients with UC from 2013, Machiels et al. found that UC does not share the microbiota signature of CD. UC patients showed a decrease in numbers of *Roseburia hominis* and *Faecalibacterium prausnitzii* relative to the control patients, as well as a decrease in short chain fatty acids (SCFAs) [71]. In 2013, Rajilić-Stojanović et al. found that, even during remission periods of UC, the dysbiosis persists. Thanks to a microarray that detects and quantifies more than 1000 intestinal bacteria, they found a decrease in numbers of the *Clostridium* cluster IV and the bacteria related to the butyrate and propionate metabolism such as *Ruminococcus bromii*, *Eubacterium rectale*, *Roseburia* sp. and *Akermansia* sp. They also found an increase in opportunistic bacteria such as *Fusobacterium* sp., *Peptosterptococcus* sp., *Helicobacter* sp., *Campilobacter* sp. *and Clostridium difficile* [72].

Dysbiosis consistently shows a decrease in SCFAs such as acetate, propionate and butyrate. These SCFAs are the primary energy source for the epithelial colonic cells [73] and promote the expansion of regulatory T cells in the colon. This decrease in SCFAs has been related to damage to the colonic epithelial cell, causing thinning of this layer, which in turn can cause diarrhoea, colitis and even pouchitis.

## 4. Inflammation in Inflammatory Bowel Disease

In a physiological way CD103+ DC induce T-reg to express gut-homing markers α4β7 and chemokine receptor type 9 (CCR9), which allow them to localize where the immune response is needed in terms of specific tissues [74,75,76]. This enhanced lymphocyte expression of gut-homing molecule α4β7 results in an alteration in the lymphocytary traffic, which is also observed in IBD [77,78,79].

After pathogenic microorganisms activate macrophages and DC, they produce IL-1b, IL-6, IL12 and IL-23 and thus activate T helper 17 (TH17) cells, γδ T cells, natural killer (NK) cells, natural killer T (NKT) cells and group 3 innate lymphoid cells (ILC3s). When these cells are activated, they will secrete tumour necrosis factor-alpha (TNF-α), interferon-gamma (IFN-γ), IL-17 and IL-22 that will stimulate intestinal epithelium to produce antimicrobial peptides (AMPs). These peptides will help secrete CXC-chemokine that is a chemo-attractant for neutrophils, which will be attracted to a specific location to produce and release ROS [80,81,82]. Out of these participating cells, both UC and CD have been uniquely related to a particular type of NK lymphocytes producing IL-22, a mediator cytokine in the response to bacteria in epithelial cells and regulator of autoimmune response. It has also been widely reported that in UC the response occurs preferentially by Th2 while in CD, Th1 is the most present cell, making the cytokines profile on both diseases vastly different [83,84]. In CD the presence of IL-1a, IL-1b, IL-12, INF-γ is imminent, while in UC IL-13 is the most abundant [85,86]. (See Table 1).

With regard to the patient’s genetic predisposition, there is evidence suggesting that individuals presenting mutations in some of the genes involved in the immune response could be more prone to the activation via NFκB for the overproduction of pro-inflammatory cytokines as IL-1b, IL-6, IL-8 and TNF [87].

These mutations have been identified in the genes that encode for the receptor NOD1 (CARD4) which mainly identifies the gram-negative bacteria peptidoglycan, as well as NOD2 (CARD15) which identifies bacteria muramyldipeptide (MDP) both Gram (+) and Gram (−), regulators of TLRs (OCTN, DLG5) and genes related to the autophagy in dendritic cells of the mucosal [88].

Regarding the possible participation of the autoimmune phenomena in IBD, the finding of the auto-antibodies against the proteins of the cytoskeleton, lymphocytes antigens, cardiolipin and pancreatic proteins has been reported both in UC and in CD [89]. Antibodies against glycoproteins of goblet cells, more specific to the intestine, have also been found but have been studied in a more superficial manner [90]. The antibodies that have been found in a more continuous matter in this disease are Anti-Neutrophil Cytoplasmic Antibodies (ANCA) and Anti-*Saccharomyces Cerevisiae* Antibodies (ASCA) predominantly [83,91].

As established, the role of a stable and healthy microbiota is very important as an inflammation inhibition mechanism and therefore crucially inhibiting IBD’s physiopathology. Clearly, intestinal microbiota exercises an important inhibiting effect of the development of pathogenic microorganisms, which is obtained by means of competitive inhibition, the decrease of permeability at an intestinal level (*L. rhamnossus*) and chemical inhibition through the secretion of lactic acid (*L. acidophilus*) and so forth. It has even been reported that the production of short chain fatty acids on by the intestinal microbiota inhibits the production of carcinogenic molecules in the intestine (*L. plantarum*) [92].

In virtue of the previous, the re-establishment of the population balance of the intestinal microbiota is certainly very important as a corrective measure of the dysbiosis observed in IBD. In fact, based on this theory, probiotics have been proposed as a therapy to avoid IBD all along.

## 5. Probiotics

The definition of probiotics has been modified and changed over time, but the definition postulated by the World Health Organization (WHO) and accepted by the International Scientific Association for Probiotics and Prebiotics (ISAPP) is the one used today: “living microorganisms that, when administered to a host in a controlled dosage, confers important health benefits.” The knowledge and information about lactic acid fermentation on humans dates back to ancient times, for example, ancient Romans and Greeks used various recipes for fermented milk. In the 20th century, Ilia Miecznikow, a Russian scientist, immunologist and Nobel Prize winner (1907), that worked for the Pasteur Institute in Paris, started a great interest in lactic acid fermentation. Since then, changes on these probiotics have been realized, including changes on their encapsulation resulting in higher resistance, better stability and the formation of a more efficient biofilm [93].

The formation of biofilm, defined by Donlan and Costerton in 2002 as “a sessile community of bacteria characterized by cells that are irreversibly linked to a substrate or interface or among them, are embedded in a matrix of extracellular polymeric substances which have produced and exhibit an altered phenotype regarding the growth speed and the transcription gene,” is a completely substantial process [94]. An effective biofilm gives the colony the capacity to protect itself against external factors, either biotic or antibiotic, to resist changes in pH, temperature and mechanical forces and to remain for longer periods in the binding site. In other words, these resistant biofilms if built in a resistant form, may create a competition with damaging bacteria already living in the host [95,96].

Still, the capacity of formation of a resistant biofilm could be affected by mutations in the genotype of certain bacteria. For example, it has been proven that *L. rhamnosus* GG decreases its capacity to form biofilms whenever a mutation in the *luxS* gene is suffered. The make-up of the composition of the media where it is growing has also been seen to be an important factor, since it is subject to temperature changes, can result in an altered chemical make-up, the availability of nutrients can vary vastly.

Aside from the media and mutations, it has been demonstrated that the capacity of bacteria to form biofilms depends on the specific strain producing it and the quantity of bacteria. For example, *L. rhamnosus* CRL 1332 and *L. reuteri* CRL 1324 creates a biofilm that is as well-structured but the second uses a higher quantity of extracellular material to create it [97]. Still, this strain of *L. reuteri* is particularly susceptible to proteases (particularly protein kinase K) since it utilizes proteins more *than L. rhamnosus CRL* 1332 to produce the biofilm [98].

In order to resist external factors that could destroy the already produced biofilm, probiotics have proved to have the capacity to inhibit its enteric pathogens by the production of lactic acid, hydrogen peroxide and bacteriocins [99]. They also create a competitive exclusion by blocking the adhesion sites of the pathogen, competing for the nutrients and modulating the system will immunize reaching the reduction of the inflammatory response [90]. All these variables complicate the development of a stable colonization. The most extensively used bacteria considering these defence mechanisms are the lactic acid bacteria including the *Lactobacillus spp* kind, which are gram-positive bacteria and facultative anaerobic [100]. Generally, probiotic products containing specific bacteria strains are developed in different formulations, ranging from chewing gum, fermented milk and capsules. The problem is that these products end up being ineffective due to different mechanisms: (1) *Bifidobacterium longum* is the only strain that can survive in fermented milk for 2 weeks. (2) The viable bacteria capable of reaching the intestinal tract is limited, because bacteria cannot survive the low pH in the stomach. Anand et al. using *Lactobacillus fermentum* 2311 in capsules made of hydroxypropyl methylcellulose phthalate (HPMCP), demonstrated that tablet formulation containing HPMCP 55 and sodium alginate showed the property to protect the bacterial strains in acidic environments like the stomach. Also, the storage is an important process to preserve the number of viable cells [101].

In conclusion, the use of probiotics has changed overtime, while new technologies for the alteration and use of probiotics are being developed. At the beginning simple forms of bacteria were used that were contained in dairy products only [102]. After observing these natural forms of probiotics were not as effective, new encapsulating techniques have been developed in order to produce bacteria that could effectively reach its action sites and produce biofilms where needed [103,104,105].

## 6. Use of Probiotics in Inflammatory Bowel Diseases

Thanks to the capability witnessed of probiotics to modulate the bacterial colonization and prevent the potentially pathogenic bacterial overpopulation, an extensive network of treatments have been used to treat gastrointestinal diseases and reduce the negative effects of antibiotics. The mechanisms of action proposed for the therapeutic effect of probiotics against enterogenic pathogens is its capacity to stabilize the intestinal mucosa, increase secretion and improve intestinal motility. In terms of immunologic action, these also modulate the inflammatory response, increasing immunoglobulin A (IgA), microbicide factors and macrophage activity.

Specifically, for inflammatory diseases, certain lines of lactobacilli and bifidobacteria probiotics have been found to mitigate UC in mice decreasing the production of pro-inflammatory cytokines. In a study made by J. McCarthy with mice knock out (KO) for IL-10, these probiotics mitigated the UC in statistically significant amounts. These results were found to have been caused by the reduction of the secretion of pro-inflammatory cytokines. These include IL-12, transforming growth factor (TGF), INF and TNF, which were observed to decrease in the presence of probiotics such as *Bifidobacterium infantis* 35624, [106].

Lammers et al. in 2003 demonstrated that stimulation with isolated bacterial DNA of stool in combination with the mixture of VSL#3 probiotics (Bifidobacterium longum, Bifidobacterium breve, Bifidobacterium infantis, Lactobacillus acidophilous, Lactobacillus casei, Lactobacillus delbrueckii subsp. bulgaricus, Lactobacillus plantarum and Streptococcus salivarius) increased the production of IL-1B and IL-10 cytokines. Bacterial DNA on one hand induced a higher production of IL-1B and the production of other pro-inflammatory factors while the treatment with probiotics of VSL #3 mainly resulted in a higher production of IL-10. The production of IL-10 specifically, was seen to produce an improved response in the immune system of the intestinal mucosa, thus mitigating the IBD symptoms [107]. Zaylaa et al. used a mix of 11 *Lactobaccillus* and *Bifidobacterium*, where they found 5 strains that had high potential for the management of IBD [108]. It is important to remember something already mentioned, that there are variables both in the host environment and in the probiotic used that complicate the development of a stable colonization.

In a systematic review published by the Mexican consensus about probiotics in gastroenterology, there is enough scientific evidence to prove that probiotics are effective in the prevention of infectious, inflammatory and functional diseases of the digestive system. However, it is necessary to evaluate the strain of the microorganism to be used in each case for its administration [109]. Other therapeutic measures used in the treatment of IBD seeking the reestablishment of intestinal microbiota is the faecal microbiota transplant (FMT). This is a much more direct method in the modification of intestinal microbiota. Although the use and benefits FMT have been controversial there is scientific evidence of its real efficacy, but this will depend of each individual clinical case. Moayyedi et al. in 2015 proved the remission in 24% of patients at the seventh week of the treatment; in their research, they indicate the participation of other factors as part of the success. They mainly refer to the type of donor, the timely treatment regarding the progress of the disease, as well as the immunosuppressant treatment [110].

Most studies of the use of probiotics in the treatment of CD use patients in remission, reporting that it is possible to limit the recurrence of the disease. Historically, the most used probiotics in the treatment of CD have been *Lactobacillus* sp. such as *L. GG* and *L. johnsonii*, however, used by themselves show poor results [111]. The use of *Saccharomyces boulardii* has shown promise in the prevention of relapse. Guslandi et al. found in 2012 a 6-fold risk of recurrence y patients treated with mesalamine as opposed to those treated with *S. boulardii* and mesalamine (*p* = 0.04) [112]. In 1993, Plein et al. found similar results [113]. However, some studies such as the one by Boureille et al. in 2013 found no significant difference between the number of relapses in the *S. boulardii* and the placebo groups [114].

Steed et al. in 2010 found symptomatic improvements with the use of *Bifidobacterium longum* with inulin and oligofructose (“Synergy 1”) [115]. Fujimori et al. found in 2007 similar results with the use of *B. longum*, *Lactobacillus casei* and plantago ovata [116]. Fedorak et al. in 2014 found that VSL#3 (a mixture of 8 different probiotics: 4 strains of *Lactobacillus,* 3 strains of *Bifidobacterium* and *Streptococcus thermofilus*) decreased the amount of inflammatory citokines in the intestinal mucosal compared to placebo (*p* < 0.05). However, there was no statistical difference between the number of patients with lesions (both severe and non-severe) [117].

There is a small number of studies about the use of probiotics in IBD and most of them seem to indicate that they are of little to no use. There is, however, a lot of room for research in this area.

Dysbiosis of intestinal microbiota plays an important role in the aetiology and pathogenesis of UC, resulting in increased numbers of proinflammatory citokines. Probiotics have been gaining a lot of attention in the last years due to their different mechanisms of action by which they help to effectively induce and maintain remission in UC patients: restore the function of disturbed mucosal barrier, inhibit competition of potential pathogens, enhance intestinal barrier function, recover intestinal microbiota imbalance and improve local and systemic immunity by decreasing proinflammatory citokines and increasing anti-inflammatory citokines [118].

Even though the data of this topic is reduced, there are recent meta-analyses and clinical trials suggesting that probiotics could be used as adjuvant therapy in some UC patients. Ghouri et al. concluded that probiotics and prebiotics helped to induce and maintain remission of UC, while there was no evidence of benefits in CD [119]. Most trials considered either *E. coli* Nissle 1917 or the probiotic VSL#3. Kruis et al. concluded that *E. coli* Nissle 1917 therapy was as effective as mesalamine in maintaining remission of UC [120]. Tsuda et al., used different probiotics, being *Enterococcus faecalis, Clostridium butyricum* and *Bacillus mesentericus* showing a reduced disease activity [121]. Although there is a reduced number of large scale and randomized trials on this topic, there is a great potential of probiotics as an alternative for pharmacological therapy in UC patients.

Another mechanism by which probiotics play an important role in the therapy of UC is their ability to recover epithelial barrier integrity, either by down regulating the proinflammatory citokines involved in the pathogenesis and immune cell activation. These limitation of inflammatory signals to the epithelial barrier may help to reduce host-induced epithelium damage. These variety of mechanisms by which probiotics modulate local and systemic immunity will be of interest in the therapy of UC patients [122]. Everard et al. showed that using *S. boulardii* in antibiotic-treated mice, resulted in a faster return to pre-antibiotic levels of specific bacterial strains, including butyrate producer species [123]. In the context of UC, it may be especially helpful to recover butyrate producers. Al-Sadi et al. showed that increased TNF-α, IFN-γ and IL-23 stimulate the epithelial barrier breakdown and can be modulated with specific probiotic strains [124], including *Lactobacillus fermentum*, *Lactobacillus salivarius*, *Bifidobacterium lactis* and mixtures of *Lactobacillus* and *Bifidobacterium* species [125]. Van der Waal et al. used a mixture called Ecologic ^®^ 825 in 2019 and achieved significant lowering of symptoms in UC, bettering the quality of life [126].

Diverse strains of probiotics, including *B. breve* [127], *B. longum* [128], *L. acidophilus* [129], *B. longum infantis* [130] and *Streptococcus thermophilus* decrease IL-17 by modulating the citokines that promote Th17 responses, thus there is a downregulation of IL-17 production.

It is important to note that even though there are diverse studies showing the efficacy of probiotics as a treatment for UC, much of them relied on in vitro models rather than in vivo models. Studies have not been consistent and more larger sample sizes and randomized trials are needed to evaluate the diverse mechanisms by which probiotics could be used as a therapy for UC.

## 7. Conclusions

Although research on probiotics and their effect on gastrointestinal infectious diseases have been realized for several years, the latest studies have focused more on the effects on DCs, as well as their impact on intestinal microbiota population and their relationship with IBD. Specifically, more studies have been recently published focusing on DCs and IBD, since there is now information on T regulator lymphocytes and their involvement in the process known as tolerance, as well as reactive oxygen species and their participation in the inflammatory process and tissue damage. As a result, multiple authors are now further encouraging probiotic therapy for not only infectious diseases but also for these inflammatory diseases, since these have proven to provide an effective homeostatic regulation of the gut microbiota, protection of the intestinal mucosal integrity, a potent anti-inflammatory effect and inhibition of pathogenic microorganism colonization.

## Figures and Tables

**Table 1:** Cytokine’s profile in Crohn’s disease (CD) and ulcerative colitis (UC). IL: Interleukin, TNF: Tumour Necrosis Factor, IFN: Interferon, I: Increased, N: Normal. Information based on: [82,83,84,87].

Cytokine	CD	UC	Cytokine	CD	UC
IL-1b	I	I	IL-5	N	I
IL-6	I	I	IL-13	N	I
IL-8	I	I	IL-17	I	I
IL-12	I	N	IL-21	I	N
IL-18	I	I	IFN-γ	I	I
IL-23	I	N	IL-4	N	I
IL-27	I	N	IL-22	I	I
TNF-α	I	I			
